# On Sten Orrenius (1937–2020)

**DOI:** 10.1038/s41418-020-0594-0

**Published:** 2020-07-21

**Authors:** Boris Zhivotovsky, Pierluigi Nicotera

**Affiliations:** 1 Karolinska Institutet, Institute of Environmental Medicine, Stockholm, Sweden; 2 DZNE, Bonn, Germany

**Keywords:** Biochemistry, Autophagy

**Figure:**
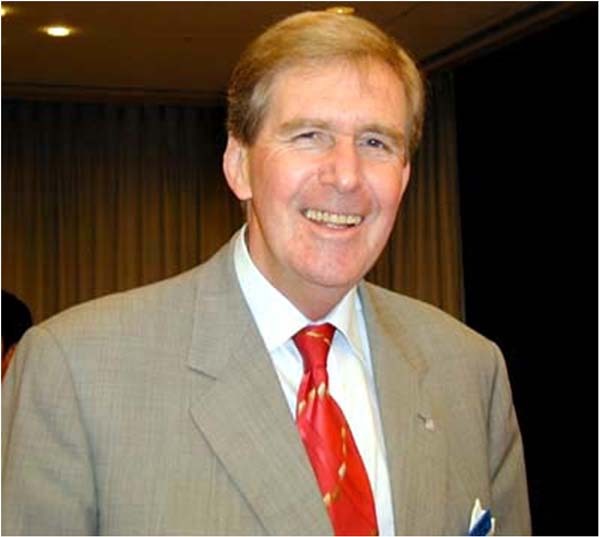
Sten’s open and warm personality and his charisma have imbued the love for Science in countless numbers of scholars.

The community of cell death researchers and the community of toxicologists lost on April 27, 2020 a highly respected scientist and a good friend Professor Sten Orrenius, “A Gentlemen”, as many of us very often called him. Sten was internationally renowned because of his groundbreaking research in drug metabolism, oxidative stress, the role of calcium in cellular toxicity and mechanisms of programmed cell death (apoptosis).

Sten was both an inspiration and a model of determination for the young generation not only because of his scientific and management achievements, but even more so for his unique personality, enthusiasm, and lifelong example. He always showed remarkable charisma, understanding of perseverance, and endurance in his character and individuality. He was always surrounded by youth, and for both young and more mature fellows it was honored to work and communicate with Sten. He was constantly full of ideas, knew all the latest news in science and generously shared them with his colleagues. Quite often Sten explained young fellows that only if they feel themselves unsatisfied, they can grow up as a researcher. Sten served with brilliance, intense energy, and effectiveness. During the whole career Sten was one of the most courteous persons not only in his own country but also worldwide.

Sten Orrenius was born on February 14, 1937 and spent his early childhood on a farm in southern Sweden. Close to the end of Sten’s study at the gymnasium, the family discussed whether he should become a farmer like his father or choose another career. Sten’s dream was to be a layer. However, before starting the University education, all young boys in Sweden were supposed to undertake 1-year military service. During that time because of a car accident, Sten changed his original plans and instead of studying law he became a medical student at the Karolinska Institutet. Importantly, medical students were able to engage in research from the beginning of their education. Also, it was possible to obtain a Ph.D. degree before finalizing the M.D. program after completion of their graduate studies. Thus, Sten was engaged at a very early stage of his career in laboratory work at the Pathology Department of the Karolinska Institutet.

Between 1961 and 1965 Sten was a Ph.D. student at the University of Stockholm under supervision of Prof. Lars Ernster. His pioneering work on cytochrome P450 was widely recognized. After his Ph.D. graduation, Sten finalized his medical education and became an M.D. He decided to pursue his career in fundamental research and started a postdoctoral training at the Department of Pharmacology at Yale University and the Department of Biochemistry at UT Southwestern Medical School at Dallas, the “Mecca” of cytochrome P450 research.

In 1971, Sten returned to Karolinska Institutet as a Professor in Forensic Medicine and focused his research on various aspects of mechanistic toxicology. He was first to introduce the use of freshly isolated hepatocytes as model for toxicology research in Sweden. Using this approach Sten’s group was able to reconstitute the overall metabolism of several drugs and xenobiotics. The detailed characterization of toxic drugs revealed not only the vital importance of glutathione conjugation in cell defense but did also indicate a role for the Ca^2+^ ion as a mediator of cytotoxicity. Since then, the latter became a focus of Sten’s research for several years. Using menadione as a model for hepatotoxicity, he showed that a combination of thiol oxidation/covalent binding and the ensuing disruption of intracellular Ca^2+^ homeostasis would lead to cell death. Further studies revealed that intracellular Ca^2+^ accumulation activated catabolic processes leading to cell death. The Ca^2+^ hypothesis of cell death was then supported over the years by observations in several pathological paradigms including heart and brain ischemia.

In 1984, Sten was appointed as a Professor in Toxicology at the Department of Toxicology at Karolinska Institutet. Studies of the role of calcium signaling in cytotoxicity connected Sten to the cell death (apoptosis) field. In these early years, of the studies on cell death mechanisms, his work highlighted the link between redox modulation, signaling, and apoptosis. The two-step concept of cytochrome *c* release discovered by Sten has obtained widespread recognition.

In collaboration with Merck, in early 90’s, Sten’s fellows established that CPP32/Apopain (now known as caspase-3) is a key interleukin 1ß-converting enzyme-like protease involved in Fas-mediated apoptosis. Sten’s group was first to describe intracellular localization and translocation of caspases and revealed some apoptotic and non-apoptotic functions of caspase-2.

Sten was also involved in early studies showing caspase-independent cell death mechanisms. As anticipated in studies in the late 80’s in hepatocytes, Sten’s group confirmed a role for calpains, the Ca^2+^-activated proteases in cell death.

Sten’s interest was focused on the crosstalk between different modes of toxic cell death. He always defended the point of view that understanding of cell death mechanisms, induced by toxic compounds, is important not only for development of drugs with limited toxicity, but also for risk assessment. Sten became one of the most cited toxicologists in the world.

Prof. Orrenius remained at Karolinska Institutet throughout his career, where he also became the founder and chair of the Institute of Environmental Medicine, a position which he held between 1988 and 1999. In 1980–1990, Sten was a member of the Senate and Faculty Board and in 1983–1990 he was Dean of the Faculty of Medicine. It was one of the most important periods at Karolinska Institutet, when a second campus was formed in Huddinge.

In addition to his many scientific accomplishments, Sten Orrenius was a member of the Nobel Assembly (1971–2003), a member of the Nobel Committee (1983–1989, 1996–2002) at Karolinska Institutet and of the Swedish Royal Academy of Sciences (since 1989). Sten received a huge number of prizes and awards, was honorary member of several academies and universities, including American Society for Pharmacology and Experimental Therapeutics, American Society for Biochemistry and Molecular Biology, Society of Toxicology (USA) and the Italian and Swedish Societies of Toxicology.

Sten was among the founders and became the first President of the European Cell Death Organization (ECDO). He was also the first recipient of the ECDO Honorary Award for Excellence in Cell Death Research. His achievements in toxicology were honored with the Distinguished Lifetime Toxicology Scholar Award by SOT.

Sten tutored many prominent scientists in the fields of toxicology and Cell Death. He was a great teacher and research mentor, inspiring big cohorts of graduate students, and postdocs, who themselves became leaders in science. In fact, more than 15 his pupils became professors at various Universities. Sten will be truly missed as a highly valued colleague, good friend, mentor, and supervisor. His scientific legacy will live forever. We would love to leave Sten with the words of his very good friend Rita Levi-Montalcini: “If I die tomorrow or in a year, it is the same—it is the message you leave behind you that counts, and the young scientists".

On behalf of the ECDO Board Pierluigi Nicotera and Boris Zhivotovsky.

